# Phonological Awareness and Nonword Repetition in Children With and Without Speech Sound Disorders: A Comparative Analysis

**DOI:** 10.1111/1460-6984.70318

**Published:** 2026-08-19

**Authors:** Özlem Icoz, Esra Özcebe

**Affiliations:** ^1^ Department of Audiology Faculty of Health Sciences Hacettepe University Ankara Turkey; ^2^ Department of Audiology Faculty of Health Sciences İstanbul‐Cerrahpaşa University İstanbul Turkey

**Keywords:** nonword repetition, phonological awareness, preschool children, speech sound disorder, verbal working memory

## Abstract

**Objective:**

This study aimed to compare phonological awareness and verbal working memory skills, as measured by a nonword repetition task, in children with speech sound disorders (SSD) and typically developing peers. It also examined the relationship between these two cognitive‐linguistic abilities within each group.

**Methods:**

The study included 30 children aged 4 to 6 years diagnosed with SSD and 30 age‐matched typically developing children. Participants were recruited from a clinical population and their typically developing siblings or relatives. Participants completed phonological awareness test and nonword repetition task. Group comparisons and correlation analyses were conducted to assess performance differences and interrelations between skills.

**Results:**

Children with SSD demonstrated significantly lower performance in both phonological awareness tasks and nonword repetition compared to the control group. Within‐group correlation analyses revealed a significant relationship between verbal working memory and word awareness in the SSD group, whereas in the control group, verbal working memory was significantly correlated with rhyme awareness.

**Conclusion:**

The findings suggest that children with SSD experience not only speech production difficulties but also weaknesses in phonological awareness and verbal working memory. The nature of the associations between these abilities appears to differ between children with and without SSD, highlighting the need for comprehensive, multidimensional assessment and intervention approaches that target both speech and underlying cognitive‐linguistic processes.

**WHAT THIS PAPER ADDS:**

*What is already known on this subject*

- Children with speech sound disorders (SSD) frequently demonstrate difficulties in phonological awareness and nonword repetition, both of which are associated with later language and literacy outcomes. Although these relationships have been widely investigated, many studies have included children with co‐occurring language impairments or have focused on English‐speaking populations. Consequently, the relationship between phonological awareness and verbal working memory in preschool children with isolated SSD remains insufficiently understood.

*What this study adds to existing knowledge*

- This study compares phonological awareness and nonword repetition performance in Turkish‐speaking preschool children with and without SSD. Children with SSD demonstrated significantly poorer performance on both measures than typically developing peers, despite age‐appropriate language abilities. The associations between phonological awareness and nonword repetition also differed between groups, providing further evidence that the relationship between phonological processing and verbal working memory may vary according to speech disorder status. The findings also contribute valuable cross‐linguistic evidence from a language with relatively transparent phonology.

*What are the actual clinical implications of this study?*

- The findings support the inclusion of phonological awareness and nonword repetition tasks alongside speech production measures in the assessment of children with SSD. A comprehensive evaluation of phonological processing and verbal working memory may facilitate earlier identification of underlying difficulties and contribute to more individualized intervention planning. Considering these cognitive‐linguistic skills together with speech production may improve clinical decision‐making and intervention outcomes.

## 1 Introductıon

Speech sound disorder (SSD) is an umbrella term that encompasses difficulties in the perception, motor production, and/or phonological representation of speech sounds, which affect speech intelligibility (Anthony et al. 2011). In other words, SSD refers to the inability to produce speech sounds that are expected to be developmentally acquired (Lewis et al. 2006b).

SSD often originates from disruptions in the phonological representations of speech segments and sounds. The ability to perceive and manipulate these speech sounds is essential for the development of accurate phonological representations (Anthony et al. 2011).

Speech and language production models suggest that both speech sound production and written/spoken language rely on a combination of shared and distinct processes. According to these models, the analysis of speech and verbal/written language is based on common phonological representations that convert either phonetic speech units or graphemes into phonemes. Two critical mechanisms supporting this conversion are phonological working memory and phonological awareness. For instance, a deficit in a shared process such as phonological working memory may negatively affect both speech production and language‐related skills, including reading and writing (Lewis et al. 2006a).

Phonological processing skills refer to a set of linguistic abilities that enable individuals to utilize their knowledge of a language's sound structure. These include metalinguistic awareness of the sound system (i.e., phonological awareness), the ability to store and immediately recall speech sound stimuli (phonological memory), and the ability to retrieve phonological representations accurately and efficiently during speech‐related tasks. These skills play a crucial role not only in speech development but also in reading and writing acquisition (Preston and Edwards 2007, Leitãto et al. 1997).

Although phonological processes can occur without conscious awareness, phonological awareness is often treated as a separate category because it involves the deliberate reflection on the phonological structure of words (Bowen 2023).

Phonological awareness is typically assessed through tasks that require children to explicitly manipulate sound structures at different linguistic levels, such as identifying rhymes, segmenting words into syllables or phonemes, blending sounds, or detecting phoneme changes within words. Performance on these tasks reflects the precision and accessibility of underlying phonological representations, which are foundational for both spoken and written language development (Stekić et al. 2023, Phillips et al. 2008)

Nonword repetition (NWR) is one of the most widely used measures of phonological working memory and provides valuable insight into children's speech processing abilities (Farquharson et al. 2021, Irizarry‐Pérez et al. 2021). According to psycholinguistic and cognitive models of verbal working memory (e.g., Baddeley 2003), NWR involves several sequential processes: accurate perceptual encoding of auditory input, phonological analysis and segmentation into phonemes, temporary storage of the sound sequence, articulatory rehearsal, and finally, the generation of a motor plan for verbal output. Within Baddeley's multicomponent model, these processes are supported primarily by the phonological loop, which consists of a phonological store that briefly retains speech‐based information and an articulatory rehearsal mechanism that refreshes these representations. Disruptions at any stage of this system may compromise nonword repetition performance. Difficulties at any of these stages—perceptual, representational, or motoric—can lead to reduced performance in NWR tasks (Baddeley 2003, Schwob et al. 2021).

Research has also demonstrated a direct association between speech accuracy and NWR performance in children with SSD. Because accurate nonword repetition requires efficient phonological encoding and the generation of precise articulatory plans, children who exhibit frequent speech errors tend to show reduced NWR accuracy. Studies indicate that phonological error patterns—particularly substitutions reflecting underlying representational weaknesses—are moderately correlated with NWR performance, whereas distorting errors show weaker associations (Farquharson et al. 2021, Brosseau‐Lapré and Roepke 2019). These findings suggest that speech accuracy and NWR draw on overlapping phonological planning and encoding mechanisms, providing a theoretical rationale for examining these domains together in SSD.

Previous research suggests that children with SSD may show reduced accuracy in NWR tasks due to deficits in phonological representations or in the phonological loop component of working memory (Farquharson et al. 2018, Tkach et al. 2011). However, the extent to which these deficits reflect limitations in phonological storage, speech‐motor planning, or perceptual encoding remains debated. While some SSD cases stem primarily from motor speech difficulties, most are considered phonological in nature, reflecting problems with phonological processing rather than articulation per se (Namasivayam et al. 2020).

SSD is a heterogeneous disorder that varies in severity, consistency, and error type (e.g., distortions vs. substitutions). Some children demonstrate primarily articulatory deficits, whereas others exhibit phonological patterning errors suggestive of underlying representational weaknesses (Namasivayam et al. 2020, Rvachew and Matthews 2024). This heterogeneity complicates interpretation of group‐level findings and underscores the importance of carefully characterizing participant profiles in research.

The relationship between phonological awareness and verbal working memory is also essential for language development. Verbal working memory plays a key role in temporarily storing unfamiliar sound sequences and integrating the phonological structure of new words into the mental lexicon (Bandini et al. 2013). In this way, it supports both language acquisition and the refinement of phonological representations during childhood. Verbal working memory is also thought to contribute to speech production processes (Adams and Gathercole 1995).

Although numerous studies have investigated working memory in populations such as children with dyslexia, developmental language disorder, or hearing impairment, fewer studies have focused on these skills in SSD (Farquharson et al. 2018, Tkach et al. 2011, Gray et al. 2019). Importantly, findings across languages are mixed. Cross‐linguistic research suggests that the manifestation of phonological awareness and nonword repetition skills may be influenced by language‐specific phonological characteristics, such as syllable structure complexity, phoneme inventory size, and morphological transparency. Languages with relatively regular phoneme–grapheme correspondences or simpler syllable structures may place different cognitive and motor demands on phonological processing tasks compared to English (Irizarry‐Pérez et al. 2021, Gibson et al. 2015). Moreover, findings across languages are inconsistent—some report clear deficits in both phonological awareness and NWR, whereas others suggest heterogeneous performance in SSD children with age‐appropriate language abilities (Brosseau‐Lapré and Roepke 2019, Stringer et al. 2024).

The overall aim of this study is to compare phonological awareness and verbal working memory skills in children with speech sound disorders and typically developing (TD) children, and to investigate the relationship between these two skills. Unlike many previous studies that have included heterogeneous SSD samples with co‐occurring language impairments, the present study specifically focused on children with SSD who demonstrated age‐appropriate language abilities, allowing a more direct examination of phonological processing independent of broader language deficits. Furthermore, phonological awareness was examined across multiple linguistic levels (rhyme, word, and phoneme awareness), and NWR performance was evaluated using both correctly repeated nonwords and correctly produced phonemes, providing a more comprehensive characterization of phonological processing than global performance measures alone. Finally, by examining these relationships in Turkish‐speaking children —a language with a relatively transparent phonological system and distinct syllable structure—this study provides data from a non‐Indo‐European language context and contributes to cross‐linguistic understanding of how language‐specific phonological properties may shape the interaction between speech production, phonological awareness, and memory processes.

## 2 Materials and Methods

Prior to the start of the study, both parents and children were provided with verbal and written information about its purpose, and written informed consent was obtained.

### 2.1 Participants

A total of 60 children aged between 48 and 72 months were included in the study. The children were recruited into two groups, each consisting of 30 participants. The study group comprised 30 children (6 girls and 24 boys) with speech sound disorders, while the control group included 30 typically developing children (7 girls and 23 boys). The average age of the children in the study group was 61.80 ± 5.01 months, while the average age in the control group was 62.66 ± 5.50 months. There was no significiant difference between the gender (*p* = 0.283) and chronological age (*p* = 0.756) between study and control group (*p* > 0.05). Participants were selected from a middle socioeconomic background, which was determined based on parental education and occupational status using a short demographic questionnaire administered prior to testing. The groups were matched based on age and gender.

Children in the SSD group were recruited from children who presented to our clinic with speech intelligibility concerns and met the study inclusion criteria. The control group consisted of typically developing children who presented to the clinic for various reasons (e.g., hearing evaluation or routine developmental assessment), as well as siblings or relatives of children attending the clinic, who met the study inclusion criteria. Initially, 68 children were screened; eight were excluded due to inconsistent participation during testing and inability to maintain adequate cooperation. None of the children in the SSD group had received prior speech or language therapy, as confirmed by parent report.

The inclusion criteria for children with study group were as follows:  1. having been diagnosed with SSD, 2. having normal language development (defined as having a standard score of 90 or above on the TEDL‐3), 3. not having any additional diagnosed or observed language and speech disorders beyond SSD (e.g., language delay, fluency disorder), 4. having bilateral normal hearing, 5. having no additional medical or developmental issues, 6. not having received any prior speech therapy, 7. Not having any craniofacial anomalies.

SSD diagnosis was based on performance on the Ankara Articulation Test (AAT) combined with detailed phonetic transcription and clinical judgment. Children who demonstrated consistent or inconsistent phonological error patterns (e.g., substitutions, omissions, phonological processes) were included in the SSD group. Children whose speech profiles were characterized predominantly by distortion errors or whose clinical presentation raised suspicion of a primary motor speech disorder (e.g., childhood apraxia of speech) were excluded from the study to reduce the influence of articulatory and motor speech confounds. The control group included children who met the following criteria:  1. having normal language development (defined as having a standard score of 90 or above on the TEDL‐3), 2. not having any diagnosed or observed language and speech disorders, 3. having bilateral normal hearing, 4. having no additional medical or developmental issues.

The methodological rationale for restricting the study group to children with age‐appropriate language abilities (TELD‐3 scores ≥ 90) was to isolate core phonological processing deficits from broader cognitive‐linguistic and morphosyntactic impairments. In clinical populations, speech sound errors and language disorders frequently co‐occur; however, including a heterogeneous sample with broader language impairments would introduce a significant confounding variable, making it difficult to determine whether deficits in phonological awareness and nonword repetition are attributable to speech‐specific representations or general language delays. Therefore, by controlling for language severity at baseline, this study aimed to investigate the integrity of phonological storage, encoding, and metalinguistic analysis specifically associated with the phonological component of speech sound disorders.

### 2.2 Test Battery

All individuals participating in the study underwent audiological evaluation and the Turkish version of TELD‐3 (Topbaş and Güven 2011). Based on the evaluation results, individuals with hearing and language development within normal limits were included in the study. All participants were also administered the Ankara Articulation Test (Ege et al. 2005), the Phonological Awareness Checklist (Akoğlu and TURAN 2011) and the Nonword Repetition List (Akoğlu and Acarlar 2014).

#### 2.2.1 Audiological Assesment

The hearing of all children included in the study was assessed through pure‐tone audiometry for both ears in IAC (Industrial Acoustics Company) soundproof rooms, using TDH39P supra‐aural headphones and a GSI‐61 clinical audiometer. A hearing screening was conducted at 500, 1000, 2000, and 4000 Hz frequencies at a 20 dB HL intensity level (Gravel et al. 1995).

#### 2.2.2 Test of Early Language Development‐Third Edition (TELD‐3)

The test, adapted into Turkish by Topbaş and Güven from the Test of Early Language Development‐Third Edition (TELD‐3), assesses the receptive and expressive language development of children aged 2;0 to 7;11 years (Topbaş and Güven 2011). The test was administered by the researcher in a quiet room, in accordance with the test's administration guidelines. Children whose receptive and expressive language development was consistent with their chronological age, based on the test results, were included in the study for both groups.

#### 2.2.3 Ankara Articulation Test (AAT)

The Ankara Articulation Test (AAT) is a standardized assessment tool developed to identify articulation problems in children aged 2 to 12 years. In addition to articulation accuracy, the test provides information about children's phonological skills. The AAT is a valid and reliable instrument with established normative data for Turkish‐speaking children. It consists of 47 colored pictures used to elicit the naming of 53 target words and evaluates 19 of the 20 consonants in Turkish (Ege et al. 2005, Ege 2010).

AAT was used in this study to both identify the speech sound problems in the study group and to determine whether the errors in the NonWord Repetition test were due to articulation/phonology issues in children with SSD as well as typically developing children.

In addition, transcription‐based error analyses were performed to document predominant error patterns (e.g., substitutions, omissions) and overall intelligibility ratings. This ensured that motor‐based difficulties were not misclassified as phonological impairments.

#### 2.2.4 Phonological Awareness Skills Checklist

To evaluate phonological awareness skills within the specific phonotactic framework of the Turkish language, the Phonological Awareness Skills Checklist (PASC) developed by Akoğlu and Turan (2011) This tool was specifically selected as a well‐regarded, targeted framework for assessing early metalinguistic abilities in Turkish‐speaking preschool populations. The PASC evaluates skills across three sections: 1. **Rhyme Skills** (5 items), assessing matching, differentiating, and maintaining rhyming words; 2. **Word Awareness** (4 items), examining structural completion and word/syllable segmentation; and 3. **Phoneme Awareness** (7 items), analyzing initial/final phoneme identification, sound blending, and segmenting.The content validity of the checklist was established during its development phase through the consensus of nine independent experts in the field, with items refined based on their feedback. Additionally, pilot applications with preschool children ensured the clarity, developmental appropriateness, and comprehensibility of the tasks (Akoğlu and TURAN 2011). During administration, children were presented with visual and auditory stimuli and asked to complete metalinguistic tasks such as identifying rhyming pairs, segmenting words, or blending sounds. Each correct response received a score of one point.

#### 2.2.5 Non‐Word Repetition Test (NWR)

This test is designed to assess verbal working memory skills in children aged 3 to 9 years. The Non‐Word Repetition (NWR) list includes a total of 36 words: 8 one‐syllable words, 8 two‐syllable words, 8 three‐syllable words, 9 four‐syllable words, and 3 words containing consonant clusters. The 36 nonsense words encompass a total of 210 phonemes. The stimuli were systematically constructed to reflect the natural syllable structures and phonotactic characteristics of the Turkish language, ensuring that vowels and consonants are equally represented across word‐initial, word‐medial, and word‐final positions. The syllable configurations of the nonwords vary systematically using V, CV, CVCC, and CVC patterns (where V represents a vowel and C represents a consonant). The nonword stimuli were professionally recorded by a male speaker in a studio environment and digitized, with a standardized 4‐second pause inserted after each word to allow sufficient time for child repetition (Akoğlu and Acarlar 2014).

During the administration of the Non‐Word Repetition (NWR) test, to mitigate potential negative effects of environmental noise from the laptop and the surrounding area, sound‐isolating headphones (model no: Sennheiser HD200) were used. Before starting the test, participants were asked to repeat nonsense words spoken by the researcher as a trial, and only those who could repeat at least 2 out of 3 words were included in the study. During the test, the words repeated by the children were recorded in the NWR recording form and simultaneously recorded using a Sony ICD‐PX240 digital voice recorder. The recordings were listened to twice by a researcher with normal hearing, and transcripts were prepared.

Two scoring methods were calculated: 1. total number of correctly repeated nonwords, which served as the primary indicator of nonword repetition performance, reflecting combined perceptual, memory, and phonological processing demands; and 2. total number of correctly produced phonemes, which was used as a supplementary measure to examine segmental accuracy and phonological encoding demands under increasing memory load, rather than as a direct index of speech motor accuracy. This secondary measure was included to explore whether reduced nonword repetition performance was accompanied by increased phoneme‐level errors, without assuming a one‐to‐one correspondence between phoneme accuracy and motor speech ability.

## 3 Statistical Analysis

The statistical analysis of the data for this study was conducted using IBM SPSS Statistics 20 software. Differences between groups in terms of age and gender were determined using the Pearson Chi‐Square Test. The normality of data distribution was assessed using the Shapiro‐Wilk Test, and the Mann‐Whitney U Test was employed for comparing non‐normally distributed data between groups. Spearman Correlation analysis was used to determine the relationship between phonological awareness skills and verbal working memory performance in both the study and control groups. Group differences in phonological awareness and nonword repetition were examined using analysis of covariance (ANCOVA), controlling for expressive language standard scores (TELD‐3) as a covariate.*p* < 0.05 indicated statistical significance.

## 4 Results

The TEDL‐3 scores of the Study and Control Groups were compared, revealing higher receptive and expressive language scores in the Control Group. While the receptive language difference was not significant (*p* > 0.05), the expressive language difference was significant (*p* < 0.05). The results are presented in Table 1.

**TABLE 1:** Comparison of receptive and expressive language scores between the study and control groups.

Language domain	Study group (*n* = 30) $\bar{X}$± SS	Control group (*n* = 30) $\bar{X}$± SS	*p*
Receptive Language	107.50 ± 9.86	109.67 ± 8.90	0.303
Expressive Language	101.97 ± 7,20	107.30 ± 8,05	**0.017**

(*p* < 0.05).

The Phonological Awareness and NWR test scores were compared between groups, and an additional analysis was conducted using ANCOVA to control for expressive language. As shown in Table 2, the SSD group demonstrated significantly lower performance than the control group across all phonological awareness subtests, with the largest group differences observed in phoneme awareness and total phonological awareness scores. Similarly, both nonword repetition measures (correct words and correct phonemes) were significantly lower in the SSD group (*p* < 0.05). These differences remained statistically significant after adjusting for expressive language (*p* < 0.05).

**TABLE 2:** Comparison of nonword repetition (NWR) and phonological awareness (PA) scores between the study and control groups.

Measure		Group	$\bar{X}$± SS	Median (Q1–Q3)	Min‐Max	*p*	*P* ^*^
Phonological Awareness	RS	Study	2.46 ± 1.22	2.5 (1.00–3.25)	1–5	**0.006**	**0.013**
		Control	3.53 ± 1.45	3.5 (2.0–5.0)	1–6		
	WA	Study	1.06 ± 0.78	1.0 (0.75–2.00)	0–3	**0.001**	**0.001**
		Control	2.53 ± 0.68	3.0 (2.0–3.0)	1–3		
	PA	Study	0.13 ± 0.34	0.0 (0.00 – 0.00)	0–1	**0.002**	**0.029**
		Control	0.93 ± 1.36	3.0 (2.0‐3.0)	0–5		
	TPA	Study	3.70 ± 1.68	3.0 (2.75–5.00	1–7	**0.001**	**0.001**
		Control	7.00 ± 2.74	7.0 (5.0‐9.0)	2–13		
Non‐word Repetition	NWR‐CW	Study	16.70 ± 4.46	16.5 (13.75–19.25)	7–26	**0.001**	**0.001**
		Control	24.46 ± 3.46	24 (22.00 – 27.00)	17–30		
	NWR‐CP	Study	179.03 ± 13.5	181.5 (170.25–189.5)	139–197	**0.001**	**0.001**
		Control	194.5 ± 5.89	195 (191.00–198.25)	180–205		

*p* < 0.05.

Abbreviations: NWR‐CP, Nonword Repetition‐ Correctly Repeated Phonemes; NWR‐CW, Nonword Repetition‐ Correctly Repeated Words; PA, Phoneme Awareness; RS, Rhyme Skills; TPA, Total Phonological Awareness Score; WA, Word Awareness;

*p* ^*^ = *p*‐value calculated after controlling for the effect of the expressive language standard score.

Within‐group correlation analyses were conducted to explore the relationship between Phonological Awareness and NWR. In the study group, a moderate positive and statistically significant correlation was found between word awareness and nonword repetition performance (*r* = 0.541, *p* = 0.002). In the control group, a moderate positive and statistically significant correlation was observed between the total phonological awareness score and nonword repetition (*r* = 0.524, *p* = 0.004). Furthermore, rhyme awareness in the control group showed a strong positive correlation with nonword repetition (*r* = 0.688, *p* < 0.001) (Table 3).

**TABLE 3:** Correlations between phonological awareness (PA) and nonword repetition (NWR) scores in the study and control groups.

		RS	WA	PA	TPA
Control Group	NWR‐CW	**0.688** ^**^	0.214	0.189	**0.524** ^**^
Study Group	NWR‐CW	0.182	**0.541** ^**^	−0.238	0.354

**p <.01.

Abbreviations: NWR‐CW, Nonword Repetition‐ Correctly Repeated Words; PA, Phoneme Awareness; RS, Rhyme Skills; TPA, Total Phonological Awareness Score; WA, Word Awareness;

## 5 Discussion

This study compared the phonological awareness and verbal working memory performance—measured via nonword repetition—of children with SSD and typically developing (TD) peers, while also exploring the relationship between these two cognitive‐linguistic skills. The findings revealed significant between‐group and within‐group differences, providing insights into the broader cognitive‐linguistic profiles of children with SSD.

Children in the SSD group scored significantly lower than their TD peers across all subtests of phonological awareness, particularly in word and phoneme awareness. These results suggest that children with SSD may have weaker cognitive representations of the sound structure of language, which negatively affects their performance in phonological awareness tasks. This aligns with previous research indicating that deficits in phonological representations may underlie difficulties in phonological awareness among children with SSD (Gillon 2017, Peterson et al. 2009). Importantly, because the present study included only children with SSD who demonstrated age‐appropriate receptive and expressive language abilities, these findings suggest that reduced phonological awareness cannot be attributed solely to broader language impairment. Rather, they support the view that weaknesses in phonological representations may be evident even in children whose general language skills fall within normal limits. This finding contributes to the ongoing discussion regarding whether phonological processing deficits represent a core characteristic of at least a subgroup of children with SSD, independent of co‐occurring language difficulties.

The SSD group also performed significantly worse on the nonword repetition task, both in terms of the number of correctly repeated words (NWR‐CW) and the number of correctly produced phonemes (NWR‐CP). Given that nonword repetition involves multiple cognitive processes—including speech perception, phonological encoding, temporary storage, and articulatory planning—deficits may arise from limitations at different stages of this processing sequence (Baddeley 2003). The present findings do not allow for conclusions regarding which specific component primarily accounts for reduced performance in the SSD group. Consistent with previous research (Roepke 2024), the present results further confirm that children with SSD demonstrate weaker nonword repetition performance compared to their typically developing peers, suggesting that phonological memory–related processes may represent an area of vulnerability in this population. Future studies incorporating production‐independent memory tasks and complementary perceptual and motor assessments are needed to better delineate the sources of NWR difficulty in this population. From a clinical perspective, these findings suggest that reduced nonword repetition performance should not be interpreted solely as a consequence of inaccurate speech production. Instead, poorer performance may also reflect vulnerabilities in phonological encoding and temporary phonological storage, emphasizing the importance of considering broader phonological processing abilities during clinical assessment. Within‐group correlation analyses showed that phonological awareness and nonword repetition performance were significantly associated in both the SSD and TD groups, although the specific subcomponents involved differed. However, the interpretation of these correlation matrices must be approached with strict caution due to severe measurement limitations. In the SSD group, the correlation between phoneme awareness and nonword repetition was weak and non‐significant. As a statistical consequence of the pronounced floor effect observed in the phoneme awareness subtest, the restricted score variability drastically truncated the covariance between variables. Because a restricted range artificially suppresses correlation coefficients, it potentially masks true clinical relationships between segmental representation and phonological working memory. Therefore, the lack of a significant correlation in the SSD group should not be interpreted as definitive evidence of independence between these cognitive systems, but rather as an artifact of statistical variance constraints induced by task difficulty ceilings. The current data do not allow for conclusions regarding which specific psycholinguistic components of nonword repetition (e.g., perceptual, phonological, or motor processes) are primarily responsible for this association. As noted by previous authors, nonword repetition reflects the combined influence of multiple processing stages, and performance differences may arise from constraints at different levels of this processing sequence. Therefore, further studies incorporating production‐independent memory measures, detailed speech perception tasks, and statistical approaches that control for articulation accuracy are needed to clarify the nature of the relationship between phonological awareness and nonword repetition in children with SSD. It is also possible that phonological awareness and verbal working memory do not develop in a fully synchronous manner during the preschool years. Different components of phonological processing may mature at different rates, particularly in children with SSD, which could contribute to the distinct correlation patterns observed across phonological awareness subdomains. Longitudinal studies are needed to examine these developmental interactions more directly.

Alternatively, these weaker correlations might also stem from the heterogeneity of SSD profiles, as children with more inconsistent or severe phonological errors could demonstrate disproportionate deficits in specific aspects of phonological awareness or memory. Future research that classifies SSD subtypes (e.g., articulation vs. phonological disorder, consistent vs. inconsistent errors) would allow for a clearer understanding of these differential relationships. Notably, the extremely low scores on the phoneme awareness subtest in the SSD group (Median = 0.0) indicate a pronounced floor effect. Phonological awareness develops in a well‐documented hierarchical sequence, moving from larger linguistic units (words and syllables) to smaller segmental units (rhymes and individual phonemes). In Turkish‐speaking populations, explicit phoneme‐level awareness typically emerges alongside formal literacy instruction around age 6 to 7. Therefore, for the preschool age cohort examined in this study (48–72 months), phoneme manipulation tasks represent a developmental ceiling, explaining the low descriptive scores even in the typically developing control group (0.93 ± 1.36). However, the near‐zero performance unique to the SSD group underscores a core deficit in establishing precise, accessible phonological representations at the segmental level. Methodologically, this restricted score variability acts as a statistical constraint that limits the variance required for robust correlation analyses, potentially obscuring true underlying associations between phoneme awareness and nonword repetition in the SSD group. Consequently, these measurement limitations necessitate caution in interpreting the lack of significant correlations in this specific subtest and highlight the need for future studies to incorporate highly sensitive, production‐independent screening tools calibrated to lower developmental thresholds for preschoolers.

The current findings suggest that children with SSD may experience difficulties not only in speech production but also in the integration of phonological awareness and verbal working memory systems. Therefore, assessment protocols for SSD should evaluate both motor and cognitive‐linguistic dimensions, and include tasks that minimize the impact of articulatory errors to better isolate underlying representational skills. In this context, production‐independent or minimally production‐dependent measures—such as the Syllable Repetition Task (SRT), which reduces articulatory complexity while maintaining phonological and memory demands—may provide a more accurate estimate of underlying phonological memory skills than traditional nonword repetition tasks (Shriberg et al. 2009).

It is noteworthy that despite both groups meeting the inclusion criteria for age‐appropriate language development (TELD‐3 ≥ 90), the SSD group exhibited significantly lower expressive language standard scores compared to their typically developing peers. This variance in expressive performance is frequently observed in clinical SSD profiles, as subtests measuring expressive language often place implicit demands on phonological production and structural organization, which may affect raw scores. While we controlled for this baseline variance using analysis of covariance (ANCOVA), the lower expressive language tendency in the SSD group might suggest a potential developmental interaction between phonological competency and expressive linguistic output. Furthermore, the fact that group differences in phonological awareness and nonword repetition remained significant after adjusting for expressive language may indicate that underlying phonological processing deficits in SSD could manifest distinctively, rather than being entirely secondary to broader expressive language proficiency.

Given that the SSD group in this study had not yet received speech therapy, the findings might also reflect the natural, pre‐intervention profile of phonological and memory‐related weaknesses, highlighting the potential for improvement through targeted remediation.

Although the present findings are broadly consistent with previous research, the study extends the existing evidence in several important ways. First, it demonstrates that weaknesses in phonological awareness and nonword repetition are evident even among children with SSD who have age‐appropriate language development, thereby reducing the likelihood that these findings are driven primarily by broader language impairment. Second, the inclusion of Turkish‐speaking children provides cross‐linguistic evidence from a language with relatively transparent phonological characteristics, contributing to the limited literature examining phonological processing in non‐English‐speaking populations. This study contributes to the literature by separately analyzing nonword repetition performance at both representational (NWR‐CW) and production (NWR‐CP) levels, allowing for a clearer understanding of the impact of speech errors on verbal memory tasks. Moreover, the separate evaluation of phonological awareness subcomponents—word, phoneme, and rhyme awareness—enabled a more nuanced analysis of their associations with memory skills. These findings highlight the need for a multidimensional diagnostic and intervention approach for children with SSD, addressing not only articulation accuracy but also the cognitive skills that support language development.

However, several limitations should be noted. Speech sound disorders constitute a heterogeneous group with variability in error types, severity, and consistency. In the present study, SSD subtypes were not analyzed separately due to sample size limitations. This may have constrained the interpretation of group‐level findings and should be addressed in future research with larger and more stratified samples. The relatively small sample size restricts the generalizability of the results. The cross‐sectional design does not allow for conclusions about developmental trajectories. In addition, verbal working memory was assessed solely through nonword repetition, limiting the exploration of other components such as semantic memory or sentence repetition. Future studies should incorporate alternative verbal memory measures that place reduced demands on speech production, such as the Syllable Repetition Task, auditory serial recall, or word learning paradigms, to better disentangle memory limitations from articulatory constraints. The low performance on certain phonological awareness subtests may have introduced floor effects, further constraining the detection of significant associations. Additionally, the exclusion of children with co‐occurring language impairments may impact the ecological validity and broader generalizability of our findings, as a substantial proportion of children seeking clinical services for SSD present with concomitant language delays. While this strict inclusion criterion was necessary to ensure high internal validity and isolate phonological processing mechanisms from general language confounds, future research should encompass more diverse clinical profiles, including children with comorbid speech and language disorders, to contrast these distinct phenotypic subgroups within the Turkish language context. Lastly, the absence of complementary assessments such as articulation or motor speech evaluations limited the ability to disentangle motor and cognitive contributions to task performance.

Future research should employ larger and more diverse samples and adopt longitudinal designs to better understand the developmental interaction between phonological awareness and verbal working memory. The inclusion of production‐independent verbal memory tasks (e.g., auditory serial recall, word learning) would also allow for a more accurate evaluation of memory abilities in children with production‐based impairments. Future studies should aim to include measures of speech perception, motor planning, and consistency of phonological errors, as well as consider age‐matched younger TD children to control for articulation‐level differences. Such studies would enhance our understanding of the cognitive profiles of children with SSD and inform the development of more comprehensive and individualized intervention programs.

## 6 Conclusion

This study demonstrates that children with speech sound disorders experience difficulties not only in speech production but also in phonological awareness and verbal working memory. These findings underscore the need for a multidimensional approach to assessment and intervention, incorporating cognitive‐linguistic components alongside speech sound targets. Future research using longitudinal designs and production‐independent tasks is needed to better understand the development of these skills and inform more effective intervention strategies.

## Funding

The authors received no specific funding for this research. The open access publication fee was covered under the TÜBİTAK–Wiley open access agreement.

## Ethics Statement

This study was conducted at Hacettepe University, Department of Audiology and Speech Disorders. It was approved by the Ethics Committee of Hacettepe University (Approval number: GO 15/79, dated 2015‐02‐04).

## Conflicts of Interest

The authors have no conflict of interest to declare.

## Data Availability

The data supporting the results reported in the manuscript are kept by the first author. Where appropriate, data analyzed or generated during the study may be shared.

## References

[jlcd70318-bib-0001] Adams, A.‐M. , and S. E Gathercole . 1995. “Phonological Working Memory and Speech Production in Preschool Children.” Journal of Speech, Language, and Hearing Research 38, no. 2: 403–414. 10.1044/jshr.3802.403 7596106

[jlcd70318-bib-0002] Akoğlu, G. , and F Acarlar . 2014. “Türkçe Anlamsız Sözcük Tekrarı Listesinin 3–9 Yaş Grubu Çocuklarda Kullanımının İncelenmesi.” Eğitim ve Bilim 39, no. 173: 13–24.

[jlcd70318-bib-0003] Akoğlu, G. , and F Turan . 2011. “Okul Öncesi Dönemde Sesbilgisel Farkındalık Eğitimi Phonological Awareness Training in Preschool Period.” Education 36, no. 161: 64–75.

[jlcd70318-bib-0004] Anthony, J. L. , R. G. Aghara , M. J. Dunkelberger , T. I. Anthony , J. M. Williams , and Z Zhang . 2011. “What Factors Place Children With Speech Sound Disorders at Risk for Reading Problems?” American Journal of Speech‐Language Pathology 20, no. 2: 146–160.. 21478282 10.1044/1058-0360(2011/10-0053)

[jlcd70318-bib-0005] Baddeley, A. 2003. “Working Memory and Language: An Overview.” Journal of Communication Disorders 36, no. 3: 189–208. 12742667 10.1016/s0021-9924(03)00019-4

[jlcd70318-bib-0006] Bandini, H. H. M. , F. H. Santos , and S. DdGd . 2013. “Levels of Phonological Awareness, Working Memory, and Lexical Knowledge in Elementary School Children.” Paidéia (Ribeirão Preto) 23: 329–338.

[jlcd70318-bib-0007] Bowen, C. 2023. Children's speech sound disorders: John Wiley & Sons;.

[jlcd70318-bib-0008] Brosseau‐Lapré, F. , and E Roepke . 2019. “Speech Errors and Phonological Awareness in Children ages 4 and 5 Years With and Without Speech Sound Disorder.” Journal of Speech, Language, and Hearing Research 62, no. 9: 3276–3289. 10.1044/2019_JSLHR-S-17-0461 31433730

[jlcd70318-bib-0009] Ege, P. 2010. “Türkçe'deki Ünsüzlerin Edinimi: Bir Norm Çalismasi.” Türk Psikoloji Dergisi 25, no. 65: 16.

[jlcd70318-bib-0010] Ege, P. , F. Acarlar , and F Turan . 2005. Ankara Artikülasyon Testi el kitabı. Ankara Üniversitesi Yayınları..

[jlcd70318-bib-0011] Farquharson, K. , T. P. Hogan , and J. E Bernthal . 2018. “Working Memory in School‐age Children With and Without a Persistent Speech Sound Disorder.” International Journal of Speech‐Language Pathology 20, no. 4: 422–433. 28306339 10.1080/17549507.2017.1293159 PMC5754259

[jlcd70318-bib-0012] Farquharson, K. , T. P. Hogan , and A. B Fox . 2021. “Factors That Influence Non‐Word Repetition Performance in Children With and Without Persistent Speech Sound Disorders.” International Journal of Language & Communication Disorders 56, no. 6: 1218–1234. 34415090 10.1111/1460-6984.12663

[jlcd70318-bib-0013] Gibson, T. A. , C. Summers , E. D. Peña , L. M. Bedore , R. B. Gillam , and T. M Bohman . 2015. “The Role of Phonological Structure and Experience in Bilingual Children's Nonword Repetition Performance.” Bilingualism: Language and Cognition 18, no. 3: 551–560. 36157200 10.1017/s1366728914000248 PMC9495270

[jlcd70318-bib-0014] Gillon, G. T. 2017. Phonological awareness: From research to practice: Guilford Publications;.

[jlcd70318-bib-0015] Ad Hoc Committee on Screening for Hearing Impairment, Handicap, and Middle Ear Disorders , Gravel, J. S. , J. Marttila , M. A. Nerbonne , et al. 1995. “Report on Audiologic Screening.” American Journal of Audiology 4, no. 2: 24–40.

[jlcd70318-bib-0016] Gray, S. , A. B. Fox , S. Green , et al. 2019. “Working Memory Profiles of Children With Dyslexia, Developmental Language Disorder, or Both.” Journal of Speech, Language, and Hearing Research 62, no. 6: 1839–1858. 10.1044/2019_JSLHR-L-18-0148 PMC6808376 31112436

[jlcd70318-bib-0017] Irizarry‐Pérez, C. D. , E. D. Peña , and L. M Bedore . 2021. “Phonological Predictors of Nonword Repetition Performance in Bilingual Children.” Journal of Communication Disorders 94: 106156. 34555787 10.1016/j.jcomdis.2021.106156

[jlcd70318-bib-0018] Leitãto, S. , J. Hogben , and J Fletcher . 1997. “Phonological Processing Skills in Speech and Language Impaired Children.” International Journal of Language & Communication Disorders 32, no. 2s: 91–111. 10.1111/j.1460-6984.1997.tb01626.x 9279429

[jlcd70318-bib-0019] Lewis, B. A. , L. A. Freebairn , A. J. Hansen , et al. 2006a. “Dimensions of Early Speech Sound Disorders: A Factor Analytic Study.” Journal of Communication Disorders 39, no. 2: 139–157. 16386753 10.1016/j.jcomdis.2005.11.003

[jlcd70318-bib-0020] Lewis, B. A. , L. D. Shriberg , L. A. Freebairn , et al. 2006b. “The Genetic Bases of Speech Sound Disorders: Evidence From Spoken and Written Language.” Journal of Speech, Language, and Hearing Research: JSLHR 49, no. 6: 1294–1312. 17197497 10.1044/1092-4388(2006/093)

[jlcd70318-bib-0021] Namasivayam, A. K. , D. Coleman , A. O'Dwyer , and P Van Lieshout . 2020. “Speech Sound Disorders in Children: An Articulatory Phonology Perspective.” Frontiers in Psychology 10: 2998. 32047453 10.3389/fpsyg.2019.02998 PMC6997346

[jlcd70318-bib-0022] Peterson, R. L. , B. F. Pennington , L. D. Shriberg , and R Boada . 2009. “What Influences Literacy Outcome in Children With Speech Sound Disorder?” Journal of Speech, Language, and Hearing Research: JSLHR 52, no. 5: 1175–1188. 19403946 10.1044/1092-4388(2009/08-0024) PMC3608470

[jlcd70318-bib-0023] Phillips, B. M. , J. Clancy‐Menchetti , and C. J Lonigan . 2008. “Successful Phonological Awareness Instruction With Preschool Children: Lessons From the Classroom.” Topics in Early Childhood Special Education 28, no. 1: 3–17. 26038613 10.1177/0271121407313813 PMC4450148

[jlcd70318-bib-0024] Preston, J. L. , and M. L Edwards . 2007. “Phonological Processing Skills of Adolescents With Residual Speech Sound Errors.” Language, Speech, and Hearing Services in Schools 38, no. 4: 297–308. 17890510 10.1044/0161-1461(2007/032)

[jlcd70318-bib-0025] Roepke, E. 2024. “Assessing Phonological Processing in Children With Speech Sound Disorders.” Perspectives of the ASHA Special Interest Groups 9, no. 1: 14–34.

[jlcd70318-bib-0026] Rvachew, S. , and T Matthews . 2024. “Considerations for Identifying Subtypes of Speech Sound Disorder.” International Journal of Language & Communication Disorders 59, no. 6: 2146–2157. 39230254 10.1111/1460-6984.13108

[jlcd70318-bib-0027] Schwob, S. , L. Eddé , L. Jacquin , et al. 2021. “Using Nonword Repetition to Identify Developmental Language Disorder in Monolingual and Bilingual Children: A Systematic Review and Meta‐Analysis.” Journal of Speech, Language, and Hearing Research 64, no. 9: 3578–3593. 10.1044/2021_JSLHR-20-00552 34407377

[jlcd70318-bib-0028] Shriberg, L. D. , H. L. Lohmeier , T. F. Campbell , C. A. Dollaghan , J. R. Green , and C. A Moore . 2009. “A Nonword Repetition Task for Speakers With Misarticulations: The Syllable Repetition Task (SRT).” Journal of Speech, Language, and Hearing Research 52, no. 5: 1189–1212. 10.1044/1092-4388(2009/08-0047) PMC2930205 19635944

[jlcd70318-bib-0029] Stekić, K. , O. Ilić , V. Ković , and A. M Savić . 2023. “ERP Indicators of Phonological Awareness Development in Children: A Systematic Review.” Brain Sciences 13, no. 2: 290. 36831833 10.3390/brainsci13020290 PMC9954228

[jlcd70318-bib-0030] Stringer, H. , J. Cleland , Y. Wren , R. Rees , and P Williams . 2024. “Speech Sound Disorder or DLD (phonology)? Towards a Consensus Agreement on Terminology.” International Journal of Language & Communication Disorders 59, no. 6: 2131–2145. 38059693 10.1111/1460-6984.12989

[jlcd70318-bib-0031] Tkach, J. A. , X. Chen , L. A. Freebairn , V. J. Schmithorst , S. K. Holland , and B. A Lewis . 2011. “Neural Correlates of Phonological Processing in Speech Sound Disorder: A Functional Magnetic Resonance Imaging Study.” Brain and Language 119, no. 1: 42–49. 21458852 10.1016/j.bandl.2011.02.002 PMC3162995

[jlcd70318-bib-0032] Topbaş, S. , and S Güven . Test of Early Language Development.: Turkish (Teld3: T). Türkce Erken Dil Gelişimi Testi (TEDİL). Detay Yayıncılık. 2011.

[jlcd70318-bib-0033] Turan, F. , and G. Akoğlu . 2011. “Okul öncesi dönemde sesbilgisel farkindalik eğitimi.” Education and Science 36, no. 161: 64–75.

